# Water absorption dynamics in medical foam: empirical validation of the Lucas–Washburn model

**DOI:** 10.1140/epje/s10189-025-00478-3

**Published:** 2025-03-05

**Authors:** Weihua Mu, Hui Sun, Lina Cao

**Affiliations:** 1 Wenzhou Institute, University of Chinese Academy of Sciences, 1 Jianlan Road, Wenzhou, 325000 Zhejiang China; 2 Wenzhou Key Laboratory of Biomaterials and Engineering, Wenzhou Institute, University of Chinese Academy of Sciences, 1 Jianlan Road, Wenzhou, 325000 Zhejiang China; 3 College of Materials Science and Opto-Electronic Technology, University of Chinese Academy of Science, Beijing, China; 4 Institute of Integrative Medicine, Department of Integrated Traditional Chinese and Western Medicine, Xiangya Hospital, Central South University, Changsha, 410008 Hunan China; 5 Center for Interdisciplinary Research in Traditional Chinese Medicine, Xiangya Hospital, Central South University, Changsha, 410008 Hunan China; 6 National Clinical Research Center for Geriatric Disorders, Xiangya Hospital, Central South University, Changsha, 410008 Hunan China

## Abstract

**Abstract:**

This study extends the Lucas–Washburn theory through non-equilibrium thermodynamic analysis to examine fluid absorption in medical foams used for hemorrhage control. As a universal model for capillary flow in porous media, the theory demonstrated strong agreement with experimental results, confirming its semi-quantitative accuracy. Minor deviations, likely due to material heterogeneity, were observed and explained, enhancing the theory’s applicability to real-world conditions. Our findings underscore the universality of the Lucas–Washburn framework and provide valuable insights for optimizing the design of medical foams, ultimately contributing to more effective bleeding control solutions in clinical applications.

**Graphic abstract:**

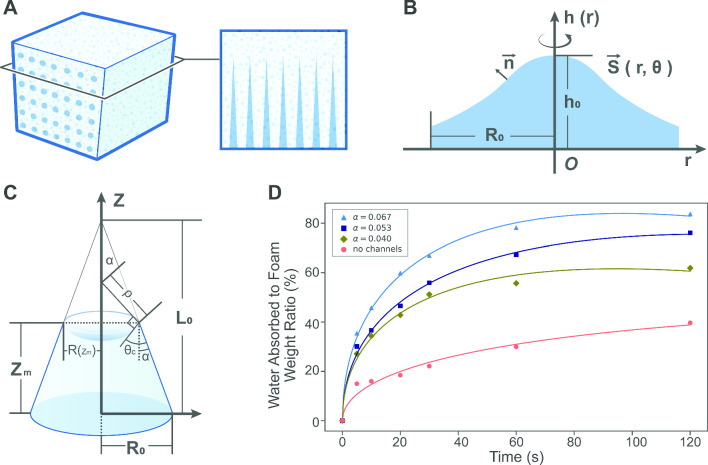

## Introduction

Acute hemorrhage remains one of the leading causes of mortality in trauma care, particularly in cases involving non-compressible wounds where traditional hemostatic techniques, such as tourniquets, are ineffective. The development of advanced hemostatic materials that can rapidly absorb blood and promote clot formation is critical to improving outcomes in emergency and surgical settings. Hemostatic foams have emerged as a promising solution due to their ability to conform to irregular wound cavities and apply localized pressure to stop bleeding [1–10].

In the use of medical foams, the kinetics of liquid absorption is crucial for their effectiveness, particularly in achieving rapid hemostasis. However, while significant research has been focused on material preparation and clinical evaluation, studies on the fundamental absorption kinetics are relatively scarce. This gap in the literature is critical, as the dynamics of fluid absorption, especially blood, are key to optimizing foam performance in emergency and surgical applications. Our work addresses this need by conducting a theoretical and experimental investigation of absorption kinetics using the well-established Lucas–Washburn rule within a non-equilibrium thermodynamic framework. By integrating material design with a detailed analysis of fluid absorption behavior, we aim to enhance the understanding of how medical foams function in real-world clinical scenarios, contributing to more effective and reliable hemostatic solutions.

The Lucas–Washburn equation, initially proposed over a century ago, has been a cornerstone in capillary flow studies. It has since been modified and extended to accommodate more complex systems, such as non-circular capillaries, heterogeneous geometries, and the influence of additional forces like gravity [11–20]. In particular, recent advancements have highlighted the importance of geometric variations in capillary-driven flows. For instance, Cencha *et al. *developed a fluid dynamic model to describe imbibition in axisymmetric closed-end pores with diameter variations along the pore depth, demonstrating that geometric changes and compressed gas back pressure significantly influence imbibition dynamics. Similarly, Reyssat *et al. *explored imbibition in channels with axial geometric variations, revealing that while short-term behavior follows the classical diffusive response, long-term dynamics deviate and exhibit power-law scaling dependent on the specific geometry. These studies underscore the critical role of geometric complexity in modifying capillary flow behavior and provide a robust theoretical foundation for interpreting fluid dynamics in non-uniform systems, such as conical channels [21, 22].

**Fig. 1:**
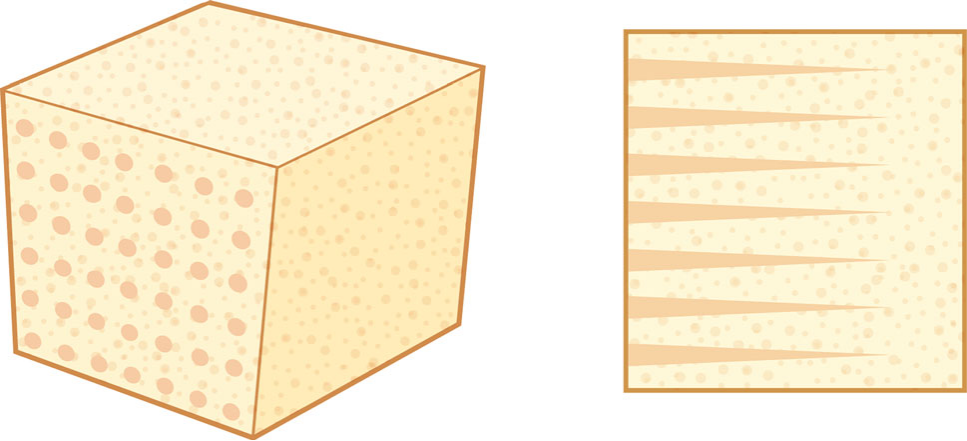
The sketch illustrates the foam structure designed with a conical channel array. The foam itself is a porous material, with conical channels featuring a fixed length of $15,{\,\textrm{mm}}$ and varying radii of $6,\,8,\,10,{\,\textrm{mm}}$. These types of foams have also been investigated for their blood absorption kinetics [23]

## Theoretical modeling

**Fig. 2:**
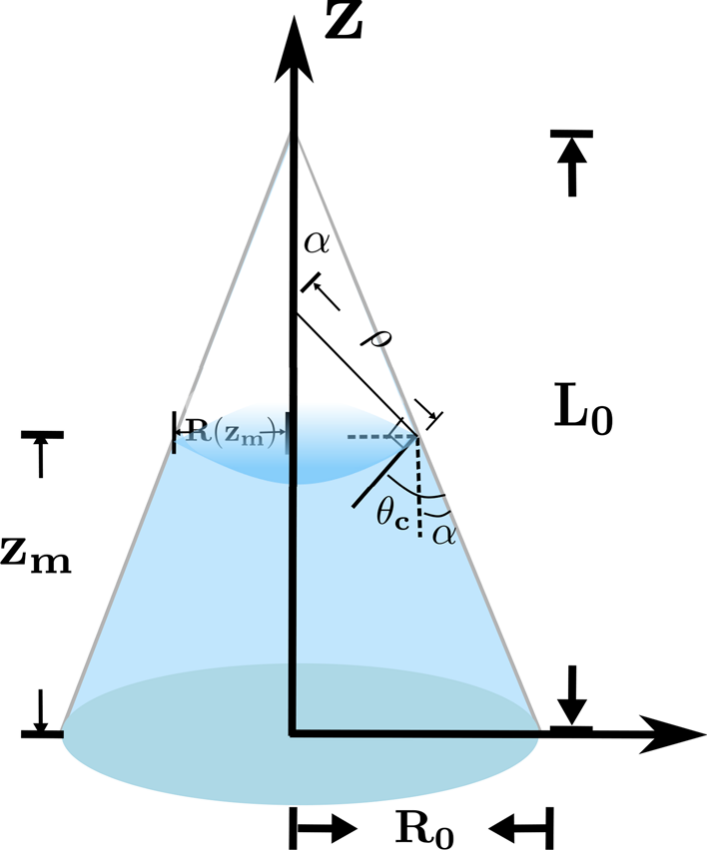
The sketch illustrates a conical channel, with the taper angle $\alpha$, the bottom radius $R_0$, the height of meniscus $z_m$, and the liquid–solid contact angle $\theta _c$

We investigate a water-absorbing sponge based on cellulose polymer materials, with artificially designed conical micro-channels embedded into the structure, providing an ideal example of capillary flow through a porous medium, as illustrated in Fig. 1 [23]. In this study, for simplicity, we focus on homogeneous micro-channel structures, beginning with a representative channel shown in Fig. 2, to analyze the liquid absorption behavior of the sponge.

The volume of absorbed water is closely linked to the rising kinetics of the meniscus within the channel, which can be understood through the framework of non-equilibrium dissipation dynamics. Various approaches to modeling these kinetics have been developed since the work of Lucas [12] and Washburn [13], as discussed in Ref. [11] and the references therein.

We subsequently demonstrate how Onsager’s principle [24], can be applied to derive the time evolution of the meniscus position $z_{0}(t)$. This principle, fundamental in non-equilibrium thermodynamics, serves as a universal variational approach that allows for the derivation of kinetic equations for systems involving energy dissipation. By employing this minimization principle, one can efficiently obtain approximate solutions to these equations.

To illustrate basic physics by a simple example, we start with the non-equilibrium theory for the process toward equilibrium. For example, with the concerning of dissipation function $F_d=L_{ij}\dot{x}_i\dot{x}_j/2$, the Lagrangian equation for a system with kinetic energy $T(\dot{\varvec{x}})$ and $V(\varvec{x})$, $L=T-V$, is modified as [25]

1 $$\begin{aligned} \dfrac{\textrm{d}}{\textrm{d}t}\dfrac{\partial {L(\dot{x}_i,\,x_i)}}{\partial \dot{x}_i}-\dfrac{\partial L}{\partial x_i} = -\dfrac{\partial F_d}{\partial \dot{x}_i}, \end{aligned}$$

with $L_{ij}$ the kinetic coefficients for the non-equilibrium phenomena, and a general flux being $J_i\equiv L_{ij}\dot{x}_j$. Famous Onsager reciprocal relations shows $L_{ij}=L_{ji}$. For the case of zero-mass system, the first term of Eq. (1) can be neglected, leading to an over-damping approximation,

2 $$\begin{aligned} \dfrac{\partial V(\varvec{x})}{\partial x_i} + \dfrac{\partial F_d( \dot{\varvec{x}})}{\partial \dot{x}_i}=0, \end{aligned}$$

For convenient, Rayleighian functional $R(\varvec{x},\,\dot{\varvec{x}})\equiv \partial _x V(\varvec{x},\,\dot{\varvec{x}})\dot{\varvec{x}}+F_d$ is introduced, and Eq. (2) has an equivalent compact form of $\partial R/\partial \dot{x}_i=0$, which is referred as Onsager’s principle in literature [24].

For the system shown in Fig. 1, to analyze the raising of the meniscus of the liquid in the converging conical tube, in the present case, we consider that the conical channel is a series of consequent straight tube pisewisely. The energy dissipation is therefore,

3 $$\begin{aligned} F_d\left[ \varvec{v}(\varvec{r})\right] =\dfrac{\eta }{2}\int \,d\varvec{r}\,\left( \dfrac{\partial v_{\alpha }}{\partial x_{\beta }}+\dfrac{\partial v_{\beta }}{\partial x_{\alpha }}\right) ^{2}. \end{aligned}$$

Assuming a Poiseuille flow in a cylindrical tube, with $\varvec{v}=v_{z}(r)\hat{e}_{z}$, and $v_{z}(r)=\left( R^{2}-r^{2}\right) \partial _z\, p/(4\eta )$, which is related to flux *Q* by $\partial _{z}p=8\eta Q/\left( \rho \pi R^{4}(z)\right)$, gets,

4 $$\begin{aligned} F_d\left[ \varvec{v}(\varvec{r})\right] \approx \dfrac{4\eta Q^{2}}{\pi \alpha ^{4}L_{0}^{3}\rho ^{2}}\int _{0}^{\tilde{z}_{m}}\,\dfrac{d\tilde{z}}{\left( 1-\tilde{z}\right) ^{4}}, \end{aligned}$$

with dimensionless quantity $\tilde{z}\equiv z/L_0$ and $\tilde{z}_m\equiv z_\mathrm{{meni}}/L_0$. The $L_0$ is the height of the conical tube, and $\alpha$ is the conical angle, and thus, the bottom radius of tube is $R_0=\alpha L_0$, and $R(z)=R_0-\alpha z=\alpha L_0\left( 1-\tilde{z}\right)$. The flux *Q* is related to the velocity of moving meniscus through $Q=\pi R^2(z_\mathrm{{meni}}) \dot{z}_\mathrm{{meni}}$,

5 $$\begin{aligned} F_d=4\eta L_{0}^{3}\left( 1-\tilde{z}_{m}\right) ^{4}\dot{\tilde{z}}_{m}^{2}\left( \tan ^{-1}\tilde{z}_{m}+\dfrac{1}{2}\ln \dfrac{1+\tilde{z}_{m}}{1-\tilde{z}_{m}}\right) . \end{aligned}$$

Next, we formulate the energy change in the liquid-foam system, accounting for the work done by surface tension and gravitational potential energy, expressed as:

6 $$\begin{aligned}  &   V\approx 2\pi \gamma \cos \theta _{c}\alpha L_{0}^{2}\int _{0}^{\tilde{z}_{m}}\,\left( 1-\tilde{z}\right) d\tilde{z}-\pi \rho g\alpha ^{2}L_{0}^{4}\nonumber \\  &   \quad \int _{0}^{\tilde{z}_{m}}\tilde{z}d\tilde{z}\left( 1-\tilde{z}\right) ^{2}, \end{aligned}$$

The kinetic equation is obtained by Onsager principle, Eq. (2), which gets,

$$\begin{aligned}  &   8\eta L_{0}^{3}\left( 1-\tilde{z}_{m}\right) ^{4}\dot{\tilde{z}}_{m}\left( \arctan \tilde{z}_{m}+\dfrac{1}{2}\ln \dfrac{1+\tilde{z}_{m}}{1-\tilde{z}_{m}}\right) \\  &   \quad +2\pi \gamma \cos \theta _{c}\alpha L_{0}^{2}\left( 1-\tilde{z}_{m}\right) +\pi \rho g\alpha ^{2}L_{0}^{4}\tilde{z}_{m}\left( 1-\tilde{z}_{m}\right) ^{2}=0. \end{aligned}$$

For the case of early stage of the kinetic process, $\tilde{z}_m\ll 1$, the kinetic equation is simplified to be:

7 $$\begin{aligned} \tilde{z}_{m}\dot{\tilde{z}}_{m}\approx -\dfrac{\pi \alpha \gamma \cos \theta _{c}}{8\eta L_{0}}\left( 1+\dfrac{\tilde{z}_m\,L_0^2}{2l_c^2\cos \theta _c}\right) . \end{aligned}$$

Here, $l_c$ is the capillary length, and $l_c\sim 2.6{\,\textrm{mm}}$ for water. The length of conical channel is $L_0=15\,{\,\textrm{mm}}$, the contact angle is about $60^{\circ }$. For $\tilde{z}_m\ll 1$, approximately,

$$\begin{aligned} \tilde{z}_{m}\dot{\tilde{z}}_{m}\approx -\dfrac{\pi \alpha \gamma \cos \theta _{c}}{8\eta L_{0}}, \end{aligned}$$

which is a simple form of L–W formula. [11]

The water is absorbed by the foam, filling both the voids in the porous material and the designed conical channels. The absorption capacity of the conical channels is significantly greater than that of the randomly distributed voids. Approximately, the volume of water absorbed by the foam can be described by:

8 $$\begin{aligned} V_{{\,\scriptscriptstyle \textrm{ab}}}=\int _{0}^{z_{\textrm{meni}}}\,dz\,\pi \,R^{2}(z)=\alpha ^{2}\pi L_{0}^{2}\left( \tilde{z}_{m}-\tilde{z}_{m}^{2}+\dfrac{\tilde{z}_{m}^{3}}{3}\right) , \end{aligned}$$

The time evolution for the weight of absorbed liquid, for the early stage, is roughly,

$$\begin{aligned} M(t)\sim a\tilde{z}_{m}-b\tilde{z}_{m}^{2}\propto c_{1}t^{{1}/{2}}-c_{2}t. \end{aligned}$$

## Experimental verifications and discussions

**Fig. 3:**
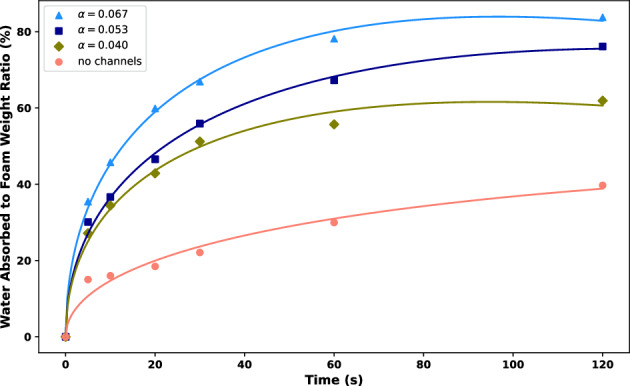
A comparative study of the theoretically predicted rule, $\Delta M/M_0 \sim a\,\sqrt{t} + b\,t$, and the experimental results are presented. The experiments involved a set of conical channels with conical angles $\alpha = 0.04,\, 0.053,\, 0.067$, alongside a channel-free sample used as a control for comparison

Compared with the experimental results, we find that the semi-quantitative predictions are in good agreement with the observations, verifying the applicability of the Lucas–Washburn rule to the water absorption kinetics in this hemostatic foam system with artificially designed micro-channels, as shown in Fig. 3.

**Fig. 4:**
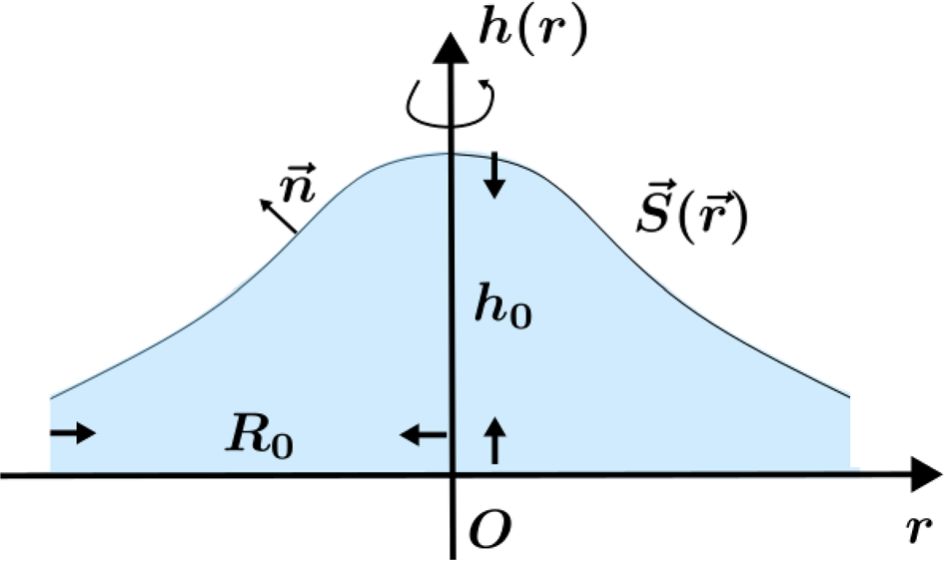
A schematic representation of the channel-free foam material after water uptake (dyed blue) illustrates the formation of a waterfront. The waterfront forms an approximately convex surface of revolution, with a higher central region and a lower periphery. For a comparative reference, see the first row of Fig. 4A in Ref. [23]

In our experiments, the water uptake behavior of the original channel-free foam material, which served as a control, exhibited a non-uniform waterfront profile. Specifically, this profile featured a higher central region and lower peripheries, as depicted in Fig. 4, which is the schematic diagram represented in Fig. 4A in our previous work  [23].

These experimental observations reveal a distinct non-uniform waterfront profile characterized by a higher central region and lower peripheries, consistent with the behavior of liquid-absorbing foam samples without channels. This phenomenon can be attributed to the spatial variation in void sizes within the foam, where the capillary pressure, $p'_c$, is inversely proportional to the characteristic void size, *d*, as described by $p'_c \propto 1/d$.

During the foam preparation process, the internal temperature gradient results in smaller voids in the core and larger ones near the surface. This gradient in void sizes leads to faster vertical accumulation of absorbed liquid in the central region compared to horizontal diffusion at the peripheries, resulting in the observed convex waterfront profile. The height of the liquid front, *h*(*r*) , at a radial distance *r* from the center of the foam can be explicitly expressed as:

$$\begin{aligned} h(r) = h_0 \sqrt{\frac{d_0}{d(r)}}, \end{aligned}$$

where $h_0$ is the reference height at the core (where $r = 0$), $d_0$ is the void size at the core, and *d*(*r*) is the void size at a radial distance *r*. Assuming a spatial distribution of void sizes following $d(r) = d_0 + \beta r^n$, with $\beta$ being a scaling parameter and *n* is an exponent describing the spatial variation of void sizes, the waterfront height *h*(*r*) can be rewritten as:

9 $$\begin{aligned} h(r) = h_0 \sqrt{\frac{d_0}{d_0 + \beta r^n}}. \end{aligned}$$

**Fig. 5:**
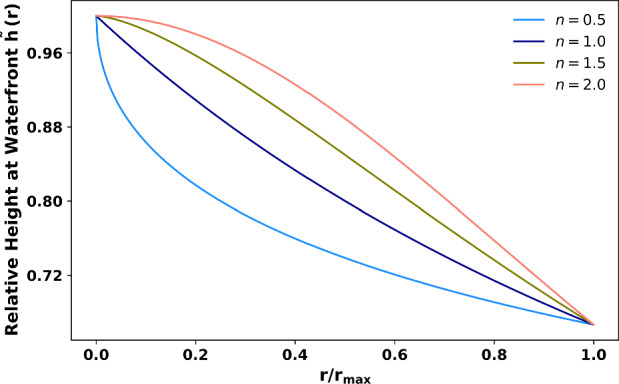
Assuming that the void size in the original channel-free foam material from the outer surface to the core follows a simple relationship given by $d(r)=d_0+\beta r^n$, the profile of the waterfront formed during water absorption in the foam sample can be accurately reproduced, as observed in the present experiment

This equation explicitly describes how the waterfront height varies with radial distance *r*, capturing the observed convex profile. For example, when $\beta = 0.1$ and $n = 2$, the waterfront height decreases monotonically from the center ($r = 0$) to the periphery ($r = R$), as shown in Fig. 5. The theoretical predictions are in good qualitative agreement with the experimental observations, validating the proposed framework.

The assumption $d(r) = d_0 + \beta r^n$ was adopted as a first-order approximation to describe the spatial variation of void sizes in the foam and its influence on the liquid absorption profile. This functional form captures the essential physics of the observed phenomenon by accounting for the experimentally evident gradient in void sizes, which results from the internal temperature distribution during foam preparation. The assumption reflects a monotonic increase in void size with radial distance, consistent with the expectation that temperature gradients produce smaller voids near the cooler core and larger voids at the warmer periphery. While this model provides a semi-quantitative explanation for the convex waterfront profile, we acknowledge its limitations in describing more complex spatial variations that could arise from non-uniform mixing or external influences during foam formation. Future studies could refine this assumption by directly measuring void size distributions using advanced imaging techniques such as micro-computed tomography (micro-CT) [26] or confocal laser scanning microscopy [27], which have been successfully employed in characterizing porous structures in soft matter and biological materials. Moreover, incorporating these experimentally obtained distributions into detailed numerical simulations could validate the model further and explore the sensitivity of liquid absorption profiles to different forms of *d*(*r*) . Such efforts would enhance the predictive capability of the model and provide deeper insights into the interplay between foam structure and liquid transport.

## Conclusion

In this work, we employed non-equilibrium thermodynamic methods to derive the Lucas–Washburn theory, widely regarded as a fundamental model for fluid transport in channels and porous media. We focused on studying capillary flow based on the balance between viscous forces and surface tension, providing a universal framework applicable to a variety of systems.

Recent advancements have extended the Lucas–Washburn framework to more complex scenarios, such as non-Newtonian fluids, irregular porous geometries, and the influence of external fields like electric fields. However, challenges persist in accurately modeling these complex systems, where the assumptions underlying the traditional Lucas–Washburn equation may no longer hold (see Ref. [11] and references therein). The universality of thermodynamic principles, such as Onsager’s principle, suggests that modifications to the conservative force energy *V* and dissipative energy $F_d$ could potentially extend the current theory to address these cases.

Our experimental investigation validated the theoretical predictions, showing that the Lucas–Washburn model accurately, and at least semi-quantitatively, describes the observed capillary flow behavior. We also identified deviations from the theoretical predictions, which we explained by considering factors such as the non-homogeneity of the medium—elements not accounted for in the original theory. Interestingly, the observed variation in the profile shape is a compelling phenomenon that invites further investigation. To gain insight into this behavior, we proposed a simplified model that qualitatively and semi-quantitatively captures the correlation between the profile shape and the curing process of the polymeric material. This model provides a useful framework for interpreting experimental observations and understanding the underlying mechanisms. However, achieving a more comprehensive understanding of this phenomenon necessitates a detailed characterization of the material’s microstructure, with a particular focus on the spatial distribution of void sizes and their evolution during curing. Ongoing work aims to address these aspects through advanced imaging and analytical techniques, which will not only refine the current model but also significantly enhance its predictive accuracy and applicability.

The Lucas–Washburn equation remains a cornerstone in capillary flow research, and future work will involve integrating advanced computational models and experimental techniques to overcome current limitations. There is growing interest in applying this theory to novel materials and emerging technologies. Continued refinement and adaptation of the Lucas–Washburn equation will enhance our understanding of capillary dynamics in increasingly complex systems.

## Data Availability

The authors declare that the data supporting the finding of this study are provided in the Appendix, where the original experimental data are presented in Table 1 along with the necessary descriptions.

## Experimental data

Table. 1 presents the experimental data on the water absorption rate of the investigated medical foam over time. The water absorption ratio is defined as the difference between the mass of the foam after water absorption and its original mass, divided by its original mass. The data include results for four types of samples: three with different channel radii and a channel-free sample serving as the control group.

**Table 1:** Experimental measurements on water absorption kinetics of the investigated medical foam with varying channel radii

Time (s)	1 mm	0.8 mm	0.6 mm	No-channel control group
0	0	0	0	0
5	0.3545	0.301	0.2715	0.15
10	0.4575	0.3665	0.344	0.16
20	0.5985	0.4654	0.4285	0.1845
30	0.669	0.5589	0.512	0.221
60	0.7815	0.6725	0.557	0.2995
120	0.8375	0.761	0.619	0.397
